# Azilsartan Increases Levels of IL-10, Down-Regulates MMP-2, MMP-9, RANKL/RANK, Cathepsin K and Up-Regulates OPG in an Experimental Periodontitis Model

**DOI:** 10.1371/journal.pone.0096750

**Published:** 2014-05-12

**Authors:** Aurigena Antunes de Araújo, Hugo Varela, Gerly Anne de Castro Brito, Caroline Addison Carvalho Xavier de Medeiros, Lorena de Souza Araújo, José Heriberto Oliveira do Nascimento, Raimundo Fernandes de Araújo Júnior

**Affiliations:** 1 Post Graduation Program Public Health/Post Graduation Program in Pharmaceutical Science/Department of Biophysics and Pharmacology,UFRN, Natal, Rio Grande do Norte, Brazil; 2 Post Graduation Program Public Health/Department of Dentistry, UFRN, Natal, Rio Grande do Norte, Brazil; 3 Post Graduation Program in Pharmacology/Department of Morphology, UFC, Fortaleza, Ceará, Brazil; 4 Department of Biophysics and Pharmacology, UFRN, Post Graduation Program in Health and Society, UERN, Natal, Rio Grande do Norte, Brazil; 5 Department of Dentistry, UFRN, Natal, Rio Grande do Norte, Brazil; 6 Department of Textile Engineered, UFRN, Natal, Rio Grande do Norte, Brazil; 7 Post Graduation Program in Functional and Structural Biology/Post Graduation Program Health Science/Department of Morphology, UFRN, Natal, Rio Grande do Norte, Brazil; Faculté de médecine de Nantes, France

## Abstract

**Aims:**

The aim of this study was to evaluate the effects of azilsartan (AZT) on bone loss, inflammation, and the expression of matrix metallo proteinases (MMPs), receptor activator of nuclear factor κB ligand (RANKL), receptor activator of nuclear factor κB (RANK), osteoprotegerin (OPG), cyclooxygenase-2 (COX-2), and cathepsin K in periodontal tissue in a rat model of ligature-induced periodontitis.

**Materials and Methods:**

Male Wistar albino rats were randomly divided into 5 groups of 10 rats each: (1) nonligated, water; (2) ligated, water; (3) ligated, 1 mg/kg AZT; (4) ligated, 5 mg/kg AZT; and (5) ligated, 10 mg/kg AZT. All groups were treated with saline or AZT for 10 days. Periodontal tissues were analyzed by histopathology and immunohistochemical detection of MMP-2, MMP-9, COX-2, RANKL, RANK, OPG, and cathepsin K. Levels of IL-1β, IL-10, TNF-α, myeloperoxidase (MPO), and glutathione (GSH) were determined by ELISA.

**Results:**

Treatment with 5 mg/kg AZT resulted in reduced MPO (*p*<0.05) and IL-1β (*p*<0.05), increased levels of IL-10 (*p*<0.05), and reduced expression of MMP-2, MMP-9, COX-2, RANK, RANKL, cathepsin K, and increased expression of OPG.

**Conclusions:**

These findings reveal that AZT increases anti-inflammatory cytokines and GSH and decreases bone loss in ligature-induced periodontitis in rats.

## Introduction

Periodontal disease is a chronic infectious, inflammatory disease of the gums and supporting tissues. Gingival inflammation that accompanies periodontal disease can damage supporting connective tissues and disrupt tooth anchoring to the jawbone. Several modulating agents have been investigated as potential therapies for periodontal disease, including antiproteinases, anti-inflammatory drugs, and bone-sparing drugs [1].

Improved knowledge of the mechanisms involved in the pathogenesis of periodontal disease have led to the use of novel agents to modulate the host response by inhibiting inflammatory mediators. Studies with antihypertensive drugs have shown that they have an anti-inflammatory activity in periodontal disease with reduced bone loss [2]–[5]. Our group have study the angiotensin II receptor blocker (ARB). For example, the angiotensin II receptor blocker (ARB) has been implicated as an anti-inflammatory agent that suppresses tumor necrosis factor (TNF)-α-induced activation of nuclear factor (NF)-κB in vascular endothelial cells [6].

Treatment of periodontitis with Telmisartan, angiotensin II receptor blocker (ARB), reduced markers of inflammation, proteases and changed proteins involved in bone remodeling [2]. Similar results were obtained in a study using another ARB, olmesartan [3].

Azilsartan medoxomil (AZT) is an ARB, approved on February 25, 2011 by the US Food and Drug Administration for hypertension management. According to the manufacturer, 4814 patients included in safety trials were treated with 20 to 80 mg azilsartan daily. Azilsartan was generally well tolerated with an overall incidence of adverse reactions similar to placebo [7]. Our group has been working with antihypertensives, angiotensin II receptor blocker (ARB), and how patients with periodontal disease have systemic diseases such as hypertension, our group aims to investigate whether these antihypertensive drugs interfere with periodontal disease. The aim of present study was to determine the efficacy of Azilsartan in treating periodontal disease.

## Materials and Methods

### Animals

Experiments were performed on male Wistar rats (180–220 g) housed in standard conditions (12 hour light/dark cycle; 22±0,1°C), with ad libitum access to food and water. All animal protocols were approved by the Animal Ethics Committee (No. 28/2012) of the Federal University of Rio Grande do Norte, Brazil. Anesthesia was induced by intraperitoneal injection of 10% ketamine (70 mg/kg, Vetnil, São Paulo, Brazil) and 2% xylazine (10 mg/kg, São Paulo, Brazil). Experimental Periodontal Disease was induced in rats under anaesthesia induced by ketamine (70 mg/kg administered i.p., 10% Quetamina, VETNIL, São Paulo) and xylazine (10 mg/kg administered i.p., 2% Calmium, São Paulo) by the placement of a sterile nylon thread ligature (3-0; Polysuture, NP45330, São Paulo) around the cervix of the maxillary left second molar [8]. Eleven days after the initial treatment, animals were euthanized with 80 mg/kg thiopental (Cristália, São Paulo, Brazil).

### Drug treatments

AZT (Edarbi, Takeda Pharmaceuticals America, USA) was dissolved in distilled water (vehicle) and administered by oral gavage 1 hour before induction of periodontitis and once daily for 10 days until euthanasia. Animals (n = 10) were assigned randomly to 5 groups: (1) nonligated, water (NL), (2) ligated, water (L), (3) ligated, treated with 1 mg/kg AZT, (4) ligated, treated with 5 mg/kg AZT, and (5) ligated, treated with 10 mg/kg AZT. Animal drug doses were not based on human dosages, because different genetic features affect pharmacokinetics of drugs. Instead, dosage was based on *in vivo* studies in rats that examined the effects of AZT on blood pressure [9], [10].

### Measurement of alveolar bone loss (ABL)

Excised maxillae were fixed in 10% neutral formalin for 24 hours, and maxillary halves were defleshed and stained with 1% aqueous methylene blue to differentiate bone from teeth. Bone loss was measured along the length of each root surface of each molar. ABL was measured in all 5 experimental groups. Three measurements were performed on the first molars (3 roots each) and 2 measurements were performed on the second and third molars (two roots each). Total alveolar bone loss was determined by taking the sum of the measurements from buccal tooth surfaces and subtracting the values of the right maxilla (no ligated control) from those of the left maxilla, in millimeters [11]. Morphometric analyses of alveolar bone were performed using standardized digital photography (Olympus SC30), and the distances (in millimeters) were measured with Imaging Software (analysis getIT 5.1).

### Histopathological analyses

Immunohistochemical analyses were performed independently by 2 oral pathologists (R.F.A. Jr. and A.A.A). Sectioning was performed in the morphology and oral pathology laboratory, and slides were analyzed using light microscopy in the Department of Morphology. Five jaws per group were used. Alveolar bone specimens were harvested, fixed in 10% neutral-buffered formalin, and demineralised in 5% nitric acid. Then, specimens were dehydrated, embedded in paraffin, and sectioned along the molars in the mesiodistal plane for hematoxylin and eosin staining. Sections (4 µm) corresponding to the area between the first and second molars where the ligature had been placed were evaluated by light microscopy (×40 magnification). Inflammatory cell influx and alveolar bone and cementum integrity were analyzed by a histologist in a single-blind fashion and graded. A score of 0 indicated that inflammatory cell infiltration was absent or sparse and was restricted to the marginal gingival region, and that the alveolar process and cementum were preserved; a score of 1 indicated moderate cellular infiltration throughout the entire gingival insert, minor alveolar resorption, and intact cementum; a score of 2 indicated accentuated cellular infiltration in the gingiva and the periodontal ligament, accentuated degradation of the alveolar process, and partial destruction of the cementum; and 3 indicated accentuated cellular infiltration, complete resorption of the alveolar process, and severe destruction of the cementum [12].

### Immunohistochemical analyses

Thin sections of periodontal tissue (4 µm) (3 jaws per group) were produced using a microtome and transferred to gelatin-coated slides. Each section was deparaffinised and rehydrated. Gingival and periodontal tissue slices were washed with 0.3% Triton X-100 in phosphate buffer, quenched with endogenous peroxidase (3% hydrogen peroxide), and incubated with the following primary antibodies (Santa Cruz Biotechnology, INTERPRISE, Brazil) overnight at 4°C: COX-2, 1∶400; MMP-2, 1∶400; MMP-9, 1∶400; RANKL, 1∶400; RANK, 1∶400; OPG, 1∶400 and cathepsin K: 1∶400. Slices were washed with phosphate buffer and incubated with streptavidin-HRP-conjugated secondary antibodies (Biocare Medical, Concord, CA, USA) for 30 minutes, and immunoreactivity to MMP-2, MMP-9, RANK, RANK-L, OPG, COX-2, and cathepsin K was visualized using a colorimetric detection kit following the manufacturer's instructions (TrekAvidin-HRP Label + Kit, Biocare Medical, Dako, USA).

### Myeloperoxidase (MPO) assay

The extent of neutrophil accumulation in gingival samples was measured by assaying MPO activity. Gingival samples (5 per group) were harvested as described above and stored at −70°C until use. Samples were homogenized and centrifuged (2000× *g* for 20 minutes), and MPO activity was determined using a colorimetric method described previously [13]. The results were reported as units of MPO per milligram tissue.

### Glutathione (GSH) assay

GSH levels in gingival tissues were measured to approximate antioxidant activity. Gingival samples (5 per group) were stored at −70°C until use. Gingival tissue homogenates (0.25 mL of a 5% tissue solution prepared in 0.02 M EDTA) were added to 320 µL of distilled water and 80 µL of 50% TCA. Samples were centrifuged at 3000 rpm for 15 minutes at 4°C. The supernatant (400 µL) was added to 800 µL of 0.4 M Tris buffer pH 8.9 and 20 µL of 0.01 M (5,5′-dithiobis-(2-nitrobenzoic acid) DTNB. Absorbance was measured at 420 nm, and results were reported as units of MPO per milligram tissue.

### IL-1β, IL-10, and TNF-α assay

Gingival tissues, stored at −70°C after extraction, were homogenized and processed as described in [14]. Levels of IL-1β (detection range: 62.5–4000 pg/mL; lower limit of detection: 12.5 ng/mL recombinant mouse IL-1β), IL-10 (detection range: 62.5–4000 pg/mL; lower limit of detection: 12.5 ng/mL recombinant mouse IL-10), and TNF-α (detection range: 62.5–4000 pg/mL; lower limit of detection: 50 ng/mL recombinant mouse TNF-α) were determined using commercial ELISA kits (R&D Systems, Minneapolis, MN, USA), as described previously [15]. All samples were measured at 490 nm.

Briefly, microtitre plates were coated overnight at 4°C with antibodies against mouse TNF-α, IL-1β, and Il-10. After plates were blocked, samples and standards were added at various dilutions in duplicate and incubated at 4°C for 24 hours. Plates were washed 3 times with buffer and antibodies were added to the wells (biotinylated sheep polyclonal anti-TNF-α, anti-IL-1β, or anti-IL-10 diluted 1∶1000 with 1% BSA assay buffer). Plates were incubated at room temperature for 1 hour, washed, and 50 µL of avidin-HRP (1∶5000) were added. The color reagent o-phenylenediamine (50 µL) was added 15 minutes later, and the plates were incubated in the dark at 37°C for 15–20 minutes. The enzyme reaction was stopped with H_2_SO_4_ and absorbance was measured at 490 nm. Values were expressed in pg/mL.

### Statistical analyses

Data are presented as means ± standard error, or as medians when appropriate. Analyses of variance and Bonferroni's tests were used to calculate the means, and the Kruskal-Wallis and Dunn's tests were used to compare medians (GraphPad Prism 5.0 Software, La Jolla, CA, USA). A *p*-value of <0.05 indicated statistical significance.

## Results

### Effects of AZT treatment on alveolar bone loss in rats with Experimental Periodontal Disease

**Figure 1:**
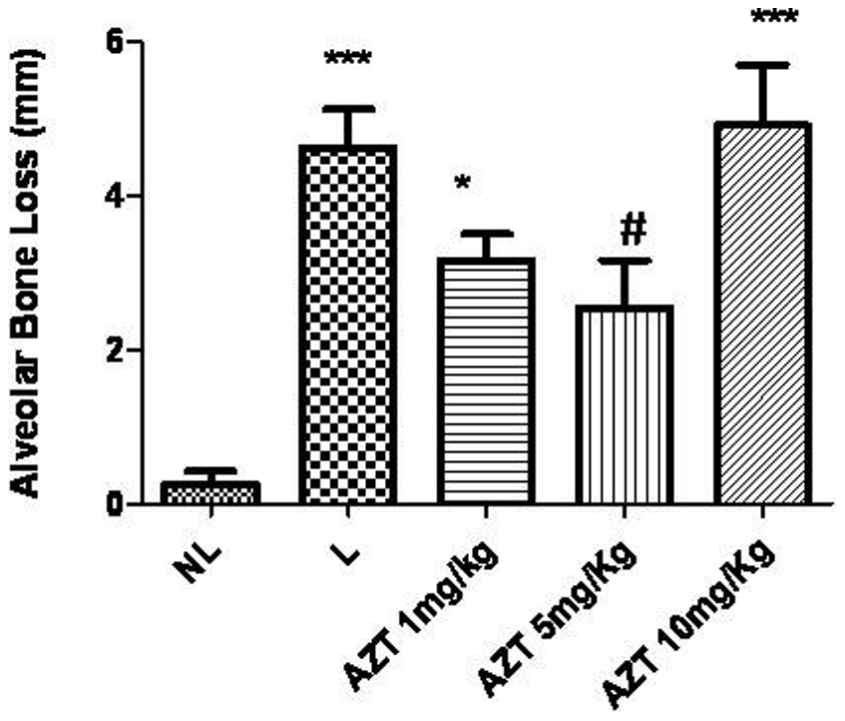
Effect of AZT treatment on alveolar bone loss associated with experimental periodontitis (EP) in rats. Values are expressed as means ±SEM (Compared to NL ***p<0.001, *p<0.05; Compared to L #*p*<0.05).

**Figure 2:**
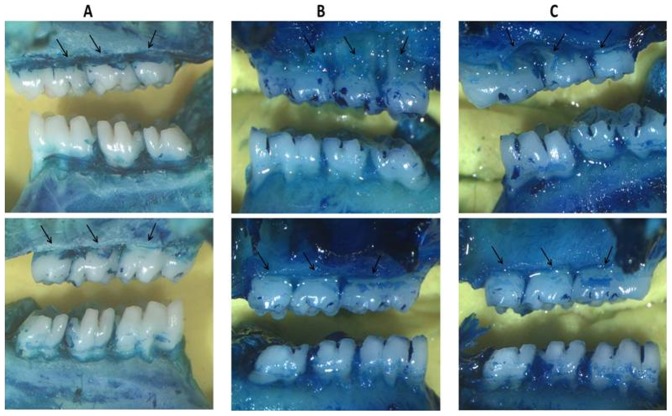
A) Superior: Left/Below: Right, NL group, showing no resorption of the alveolar bone. B) Superior: Left/Below: Right, L group, showing severe bone resorption with root exposure (arrows). C) Superior: Left/Below: Right, L group treated with Azilsartan 5 mg/kg, showing decreased bone resorption (arrows). Images were obtained at an original magnification of 1.7×.

Rats with ligation-induced periodontitis (L) showed significant alveolar bone loss compared with non-ligated animals (NL) (NL = 0.22+0.41 mm; L = 4.6±1.4 mm; *p*<0.001). AZT treatments (5 mg/kg) reversed alveolar bone loss caused by periodontitis compared to L animals (AZT 5 mg/kg 2.5±1.9; L = 4.6±1.4 mm, *p*<0.05) (Figure 1). The macroscopic aspects of the NL group with no resorption of the alveolar bone compared to the L group with severe bone resorption and root exposure can be seen in (Figure 2B). Figure 2C shows the macroscopic appearance of the periodontium after ligation and treatment with AZT 5 mg/kg, which led to decreased bone loss compared to ligated animals. Rats treated with 1 mg/kg and 10 mg/kg AZT also showed differences in alveolar bone loss compared to NL animals (AZT 1 mg/kg = 3.2±0.8 mm; AZT 10 mg/kg = 4.9±1.9 mm, *p*<0.001 and *p*<0.05, respectively) (Figure 1).

### Histological analyses

**Figure 3:**
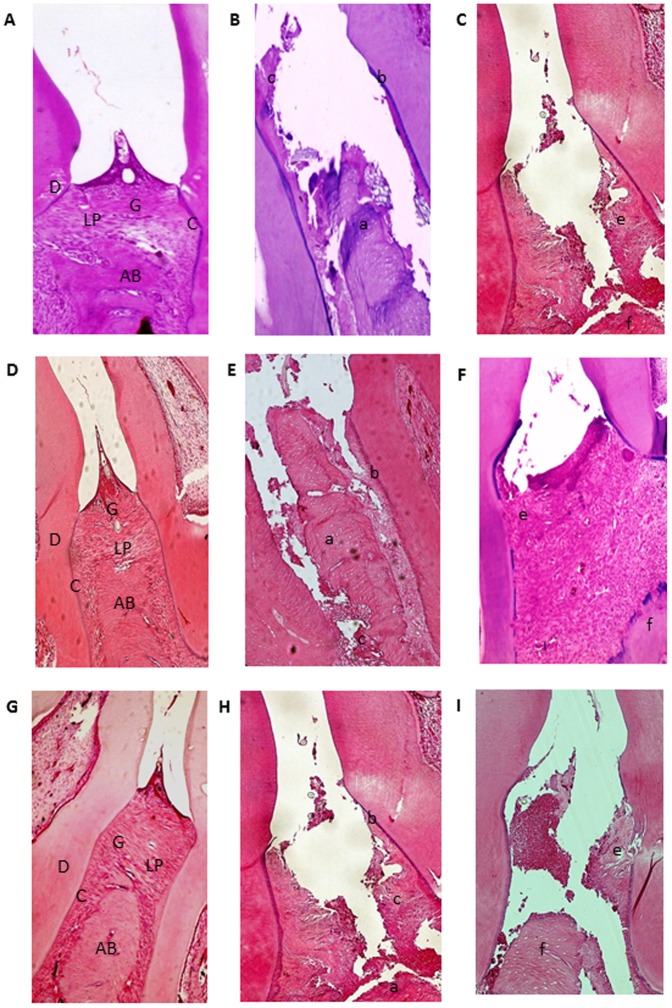
Microscopic analyses. A, D and G: Normal periodontium. B, E, H: Periodontium from a rat with periodontitis (treated with saline) showing alveolar bone and cementum resorption (discontinuous cementum) and inflammatory cell infiltration. C: Treatment with AZT (1 mg/kg) and I: Treatment with AZT (10 mg/kg) showing no reduced inflammation and increased alveolar bone loss. F: Periodontium from a rat with periodontitis (treated with AZT, 5 mg/kg) showing reduced inflammation and decreased alveolar bone loss. Sections were stained with H&E. Original magnification 40×. Scale bars  = 100 µm. G, gingiva; PL, periodontal ligament; D, dentin; AB, alveolar bone; C, cementum; a, bone loss; b, resorption of cementum; c, inflammatory process; e, f, decreased inflammation process and bone loss.

Figure 3 shows the structures of the periodontium, gingiva, periodontal ligament, alveolar bone, and cementum prior to ligation. Histopathological analyses revealed that alveolar bone loss was reduced in ligated animals treated with 5 mg/kg AZT (*p*<0.05) compared to ligated animals treated with 1 mg/kg and 10 mg/kg AZT. Discrete cellular infiltration restricted to the region of the marginal gingiva, preservation of alveolar bone, and intact cementum were observed in animals treated with 5 mg/kg AZT (Figure 3F). In animals subjected to ligation that received no treatment (L), inflammatory cell infiltration and severe destruction of the cementum and alveolar process were observed, animals in this group received median scores of 3 (Figure 3B,E,H; Table 1). Treatment with 5 mg/kg AZT prevented ligation-induced inflammation (Figure 3F) and reduced median histopathological scores 1 (1–2) (Table 1).

**Table 1:** Histological analysis of maxillae from rats presenting with periodontal disease, Natal, RN, 2014.

NL	L	Azt 1 mg/kg	Azt 5 mg/kg	Azt 10 mg/kg
0 (0-0)	3 (3-3)#	3 (3-3)#	1 (1-2)	3 (2-3)#

# p<0.05, Compared with NL.

### Immunohistochemical analyses of inflammatory markers

**Figure 4:**
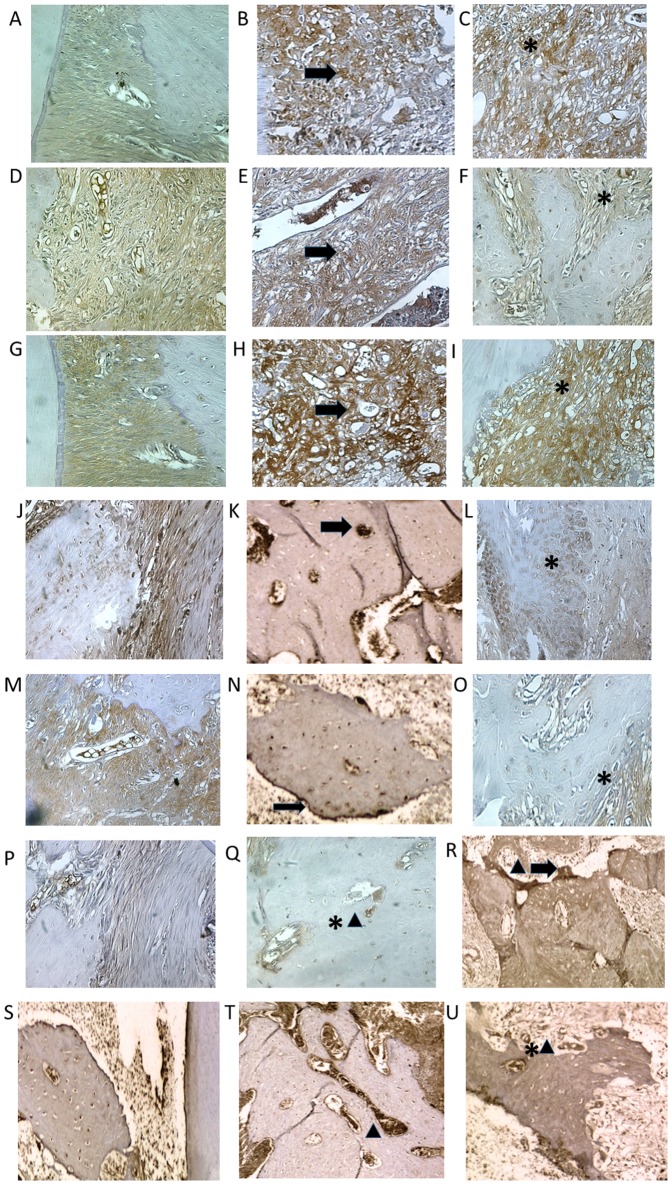
Photomicrographs of periodontal tissue of PD rats treated with AZT, showing immunoreactivity to MMP-2, MMP-9, COX-2, RANK, RANK-L, OPG, and cathapsyn. Rats subjected to saline (A, D, G, J, M, P,S); rats subjected to ligation (B, E, H, K, N, Q,T); rats subjected to ligation and treated with AZT (5 mg/kg) (C, F, I, L, O, R,U). Images are shown at 40× magnification. Bar  = 100 µm. Arrow indicates high or moderate labeling in the periodontal ligament or the alveolar bone. Asterisk indicates mild or moderate labeling in the periodontal ligament or the alveolar bone. Triangle and asterisk indicate mild labeling of OPG in osteoclasts. Triangle and arrow indicate high labeling of osteoclasts. Triangle indicates intense labeling of Cathepsin K on alveolar bone. Asterisk and triangle indicate mild labeling of cathepsin K on alveolar bone.

Compared to the NL group, the periodontium of rats in the L group showed marked immunostaining for MMP-2, MMP-9, COX-2, RANK-L, RANK, OPG and Cataphesyn (NL-saline animals, Figure 4A, D, G, J, M, P and S compared to L-saline animals, Figure 4B, E, H, K, N, Q and T). However, treatment with AZT (5 mg/kg) reduced MMP-2, MMP-9, COX-2, RANK-L, RANK, and Cataphesyn, immunostaining in the periodontium of rats subjected to ligation (Figure 4 C, F, I, L, O and U). OPG immunostaining was mild in the periodontium of the L group, moderate in the NL group, and intense in the group treated with 5 mg/kg AZT (Figure 4R).

### Effects of AZT on inflammatory activity and GSH

**Figure 5:**
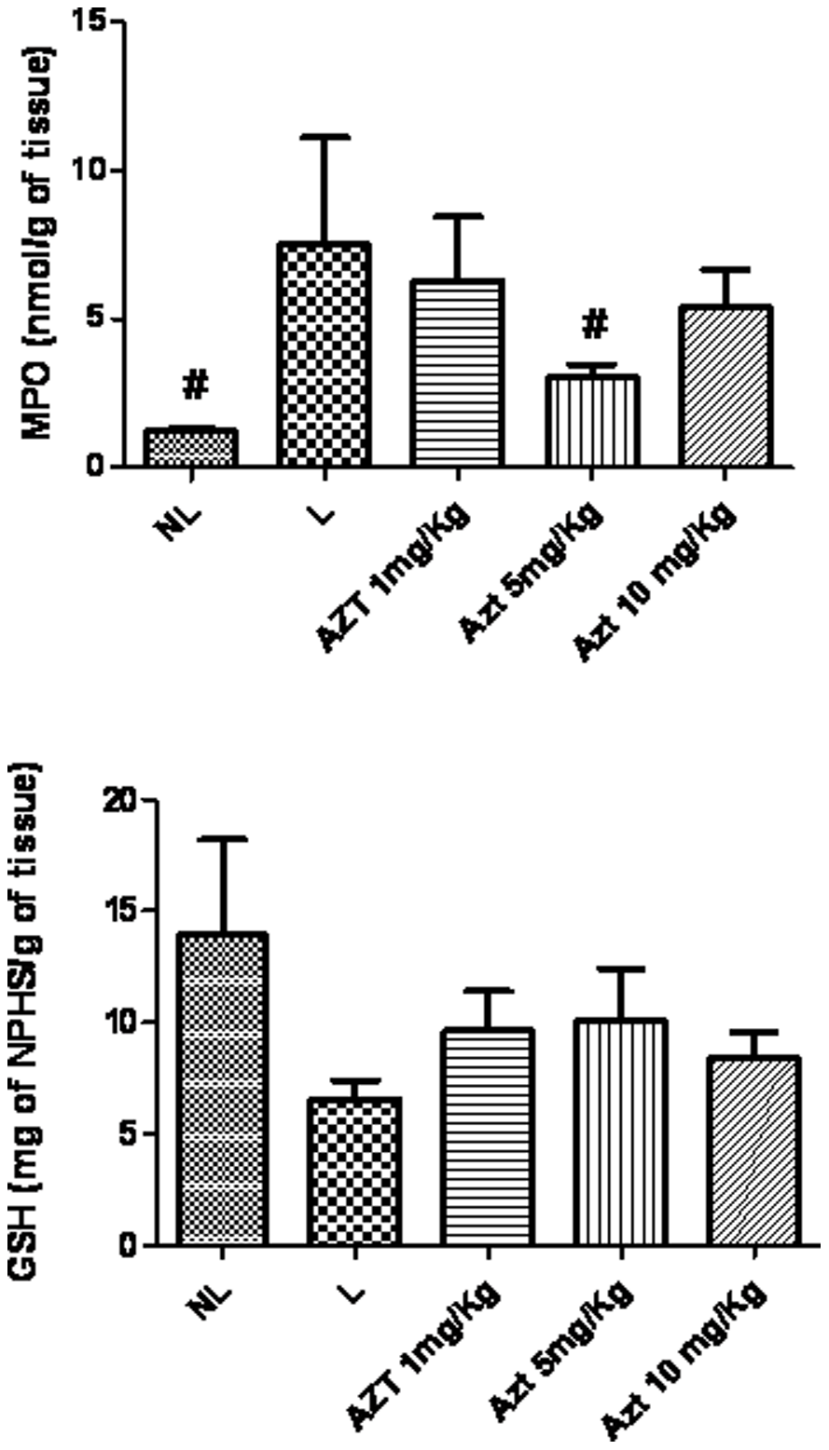
MPO and GSH in NL, L, and groups treated with 1/kg, 5 mg/kg, and 10 mg/kg AZT (*p<0.05, **p<0.01).

**Figure 6:**
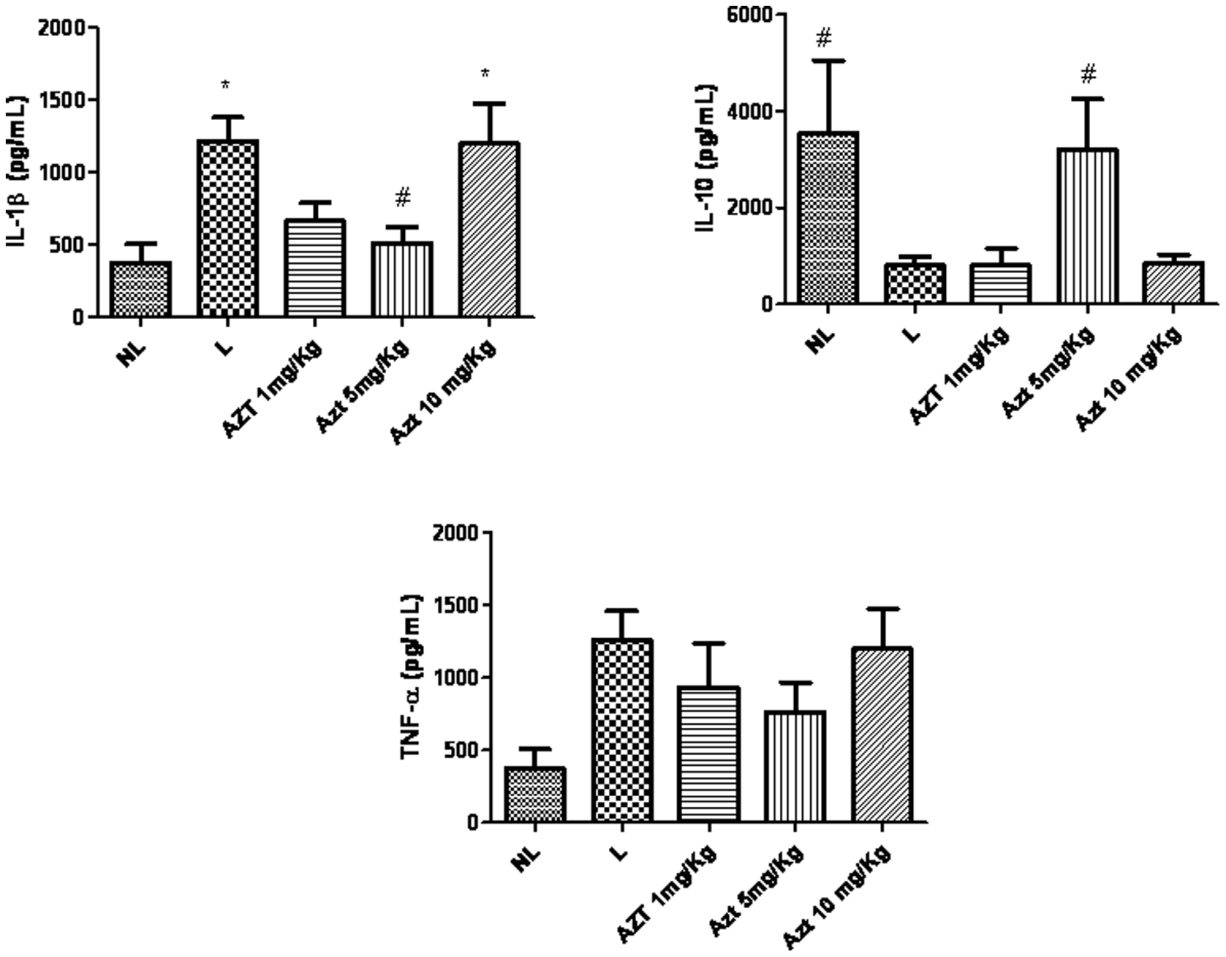
Levels of A) IL-1β, B) Il-10, and C) TNF- α, in NL, L, and AZT-treated animals (1 mg/kg, 5 mg/kg, and 10 mg/kg) (*p<0.05).

MPO activity was increased in the L group compared to the NL group (*p*<0.05). The group subjected to ligation and treated with 5 mg/kg AZT showed reduced concentrations of MPO compared to the L group (*p*<0.05) (Figure 5). Levels of antiinflammatory cytokine IL-1β were decreased in animals treated with 5 mg/kg AZT compared to L group animals (*p*<0.05). Levels of antiinflammatory cytokine IL-10 were increased in animals treated with 5 mg/kg AZT compared to L group animals (*p*<0.05) (Figure 6). The treatment of with AZT no reduced the levels of GSH (p>0.05) compared with those of the L group (Figure 5).

## Discussion

Periodontitis is a chronic inflammatory disease resulting from perturbed homeostasis between the subgingival microbiota and host defenses [16].Given the extensive use of antihypertensive drugs, combined with the fact that periodontal disease affects adults and the elderly, we decided to investigate the effect of antihypertensive drugs on the progression of periodontal disease. In this study, azilsartan treatment produced some results than previous studies by our group using other antihypertensives. Telmisartan and Olmesartan significantly reduced pro-inflammatory cytokines such as IL-1β and TNF-α, which resulted in significant preservation of bone in periodontal disease [2], [3]. In the periodontium, the activation of IL-1β and TNF-α is known to stimulate the degradation of connective tissue matrix, the activation of osteoclasts, and the resorption of bone [17], [18]. IL-1 induces the release of MMPs, whereas TNF-α, which is present in inflamed gingival tissue, is involved in the destruction of tissue [19]. These cytokines play key roles in the breakdown of periodontal tissue through collagenolytic enzymes such as MMPs. The inflamed tissue also expresses COX-2, which may play a role in the formation of arachidonic acid metabolites. In this study, MPO and IL-1β levels were significantly reduced by AZT treatment (5 mg/kg). Reduction in the inflammatory response can be confirmed by observing reduction in tissue COX-2, an enzyme expressed in inflamed tissues, that was markedly reduced in rats treated with Azilsartan (5 mg/kg) compared to levels observed in rats presenting with periodontal disease.

We believe these results may be related to IL-10 increased levels. According to a study by Gaddis *et al.* (2013), IL-10 plays an important role controlling infection and the progression of periodontal disease [20]. Surface components of *P. gingivalis*, such as LPS, lipoproteins, and fimbriae, interact with host-expressed Toll-like receptors (TLRs), key control elements of the innate immune response. TLR activation leads to nuclear translocation of NF-κB and induction of inflammation-related genes, suggesting that exaggerated inflammation mediated by TLRs is a driving factor in periodontal disease [21]. One of the most effective suppressors of TLR-induced inflammatory cytokine production is IL-10, which inhibits innate immune cells by directly inhibiting cytokine transcription [22].

The main problem related to periodontal disease is bone loss, which is caused by activation of lymphocyte pro-inflammatory cytokines, such as IL-1β and TNF-α, and may be mediated by significant increases in IL-10 levels. IL-10 can mediate bone loss through both direct and indirect actions. It inhibits bone resorption indirectly by upregulating osteoprotegerin expression and downregulating expression of RANKL [23]. IL-10 mediates bone loss directly by inhibiting osteoclast formation [24].

MMPs are a family of related zinc-containing proteinases that can degrade most of the extracellular matrix. It has been reported that the induction of MMPs (such as MMP-2) in osteoblasts is essential for bone resorption [25]. Excessive production of MMP-2 and MMP-9 can lead to accelerated matrix degradation in pathological conditions such as periodontitis [25]. Tissue destruction caused by MMP-2 and MMP-9 was reduced in animals treated with 5 mg/kg AZT, indicating that IL-10 may be a protective cytokine in periodontal diseases [26]. We also observed reduced RANKL and RANK staining and increased OPG staining. These results are corroborated by studies showing that inhibition of osteoclast formation by IL-10 is mediated through direct actions on osteoclast precursors [24], [27], including RANKL-induced osteoclastogenesis. IL-10 potently reduced the RANKL-induced expression, which are essential for osteoclastogenesis [28]. A study by Liu *et al.* (2006) suggested that IL-10 inhibits bone resorption by up-regulating OPG expression and down-regulating RANKL expression [23].

The study by Gallet *et al*. (2006) showed that expression of MMP-1, MMP-2, MMP-9, and RANKL was correlated with expression of IL-1β, TNFα, and interferon-gamma inflammatory reactions and alveolar bone loss. On the other hand, IL-4 and IL-10 were associated with higher expression of OPG, with lower expression of MMPs and RANKL, and with reduced rates of cellular infiltration in periodontal tissues and alveolar bone loss [29].

The study of Bostanci et al (2011) aimed to monitor quantitative changes in the RANKL–OPG system in gingival crevicular fluid (GCF) following non-surgical periodontal treatment over a period of 4 months. The data indicated that periodontal treatment does not affect RANKL or OPG levels in chronic periodontitis (CP) patients. Hence, conventional therapy alone cannot modify the overall capacity of the tissue to produce these factors. Levels of RANKL and OPG transiently increased in the GCF 2 months post-treatment, with RANKL changes being statistically significant [30]. Adjunctive therapies for periodontal disease management may modulate RANKL/OPG ratio [31].

Another important marker of bone is Cathepsin K, a cysteine protease that is released from mature osteoclasts to degrade type I collagen, the major organic matrix protein in bone. It is an important mediator of osteoclast activity and is relatively abundant and selective compared to other collagenases in these cells [32]. Therefore, reduction of Cathepsin K in the AZT treated group (5 mg/kg) implies that AZT treatment reduces degradation of the bone matrix.

In conclusion, this study revealed that treatment with 5 mg/kg doses of AZT reduces bone loss, decreased IL-1β, increased IL-10 and decreased MPO levels, and reduced expression of RANKL/RANK and cathepsin K. These changes increased OPG staining, which controls osteoclastogenesis. Taken together, these data indicate that AZT is an effective treatment to decrease bone loss in ligature-induced periodontitis in rats.
